# Egg buoyancy variability in local populations of Atlantic cod (*Gadus morhua*)

**DOI:** 10.1007/s00227-012-1984-8

**Published:** 2012-06-29

**Authors:** Kyung-Mi Jung, Arild Folkvord, Olav Sigurd Kjesbu, Ann Lisbeth Agnalt, Anders Thorsen, Svein Sundby

**Affiliations:** 1 Institute of Marine Research, P.O. Box 1870, Nordnes, 5817 Bergen, Norway; 2 Department of Biology, University of Bergen, P.O. Box 7800, 5020 Bergen, Norway

## Abstract

Previous studies have found strong evidences for Atlantic cod (*Gadus morhua*) egg retention in fjords, which are caused by the combination of vertical salinity structure, estuarine circulation, and egg specific gravity, supporting small-scaled geographical differentiations of local populations. Here, we assess the variability in egg specific gravity for selected local populations of this species, that is, two fjord-spawning populations and one coastal-spawning population from Northern Norway (66–71°N/10–25°E). Eggs were naturally spawned by raised broodstocks (March to April 2009), and egg specific gravity was measured by a density-gradient column. The phenotype of egg specific gravity was similar among the three local populations. However, the associated variability was greater at the individual level than at the population level. The noted gradual decrease in specific gravity from gastrulation to hatching with an increase just before hatching could be a generic pattern in pelagic marine fish eggs. This study provides needed input to adequately understand and model fish egg dispersal.

## Introduction

Egg buoyancy, Δρ, is defined as the difference in specific gravity between the ambient sea water, ρ_w_, and the specific gravity of the egg, ρ_e_, that is, Δρ = ρ_w_ − ρ_e_ (Sundby 1991). Most pelagic eggs, that is, egg with a positive buoyancy (Sundby 1983), have specific gravity that is very close to that of the sea water in which the eggs are found. Typically, Δρ in pelagic fish eggs ranges from 1 to 3 × 10^−3^ g cm^−3^. The mechanism for achieving hydrostatic lift is caused by high water content completed during the process of oocyte hydration in the ovary (Craik and Harvey 1987) while the volume of chorion material, which is the heavy fraction of the egg, reduce the buoyancy (Kjesbu et al. 1992). Stock-specific difference in egg specific gravity clearly exists between brackish water and marine cod eggs (Thorsen et al. 1996). For example, the Atlantic cod in the Baltic Sea has developed large eggs of high water content (97 %) and thin chorion (thickness: <2 μm). As a result, they are neutrally buoyant at salinities of 12.3–18.3 (Nissling et al. 1994), which make them float above the hypoxic deeper layers. However, because of the very low-saline surface layers, these eggs are bathypelagically distributed at the halocline below the surface layer. Atlantic cod eggs in the high-saline North Atlantic basin are smaller and heavier with less water content (93 %) and thicker chorion (thickness: 5–9 μm) (Lønning et al. 1984), neutrally buoyant at salinities of 29.5–33 (Solemdal and Sundby 1981). Despite these circumstances, they are pelagically distributed with highest concentrations in the surface layers because of the high-saline ambient water.

In northern Norway, Northeast Arctic cod (NEAC) and Norwegian Coastal cod (NCC) are managed as separate units. The two stocks can be distinguished from one another by differences in otolith structure in terms of growth zones and shape (Stransky et al. 2008), genetics (Sarvas and Fevolden 2005), and the number of vertebrae (Berg and Albert 2003). Also the two stocks have different life history characteristics. Adult NEAC undertake long migrations from offshore feeding grounds in the Barents Sea to spawning areas located along the Norwegian coast from Møre in the south to Finnmark in the north and with the centre of concentration in the Lofoten area (Sundby and Nakken 2008). Spawning occurs mainly during March and April (Ellertsen et al. 1989). Age and length at maturity are 6–8 years and 75–90 cm, respectively (Bergstad et al. 1987; Olsen et al. 2010). In contrast to this life history, NCC inhabit coastal areas and fjords of Norway with short migration routes. The spawning areas appear close to those of NEAC, but are generally more inshore along the coast and into the fjords. More specifically, some of the outer coastal areas overlap partly with spawning areas of NEAC, but NCC typically spawn closer to shore and shallower (Olsen et al. 2010); peak spawning occurs 3–4 weeks later than for NEAC (Nordeide 1998). However, the magnitude of this difference in timing very much depends on local temperatures in relation to those in the Barents Sea experienced during the whole length of gonad maturation (Kjesbu et al. 2010). Hence, it is expected to be considerable latitudinal change in spawning time among the various coastal and fjord populations being later in the cool north and earlier in the warmer south. Fjord-spawning NCC is more stationary than coastal-spawning NCC (Olsen et al. 2010). Both categories of NCC reach maturity at 5–6 years/40–50 cm (Berg and Pedersen 2001).

The Norwegian coastline is long and rugged with numerous fjords, so NCC are distributed over different environmental conditions, which in turn may influence population traits. Norwegian fjord systems with sills function as sheltered areas from strong currents, and favour local retention of early life stages of cod (Asplin et al. 1999). The small-scale egg retention has been found in fjords of both western (Knutsen et al. 2007) and southern Norway (Espeland et al. 2007; Ciannelli et al. 2010) associated with shallow sills and narrow entrances. However, also in the large fjords of Northern Norway with mostly very deep sills and thereby large exchange of water masses, the eggs are retained due to the combination of the vertical salinity structure, the estuarine circulation, and egg specific gravity (Myksvoll et al. 2011). Studies on local cod populations showed that they are not only genetically separated (Knutsen et al. 2003; Dahle et al. 2006; Jorde et al. 2007; Knutsen et al. 2011) but also have different spawning time (Otterå et al. 2006) along the coasts and fjords of Norway. Thus, given that environmental variances can influence life history parameters (e.g. Imsland and Jónsdóttir 2002), it is of interest to investigate whether NCC has developed special characteristics in egg specific gravity selecting for vertical distributions that would favour local retention of eggs, which in turn would contribute to local recruitment and separation of populations.

Our primary objectives are to investigate, using three local populations of NCC reared in modern aquaculture systems, (1) how maternal attributes affect egg specific gravity, (2) how seasonal (i.e. during the spawning season) and daily (i.e. during embryonic development) changes in egg specific gravity differ among these populations, and (3) how phenotypes of egg specific gravity vary among genetically differentiated families.

## Materials and methods

### Collection of cod broodstocks

**Fig. 1:**
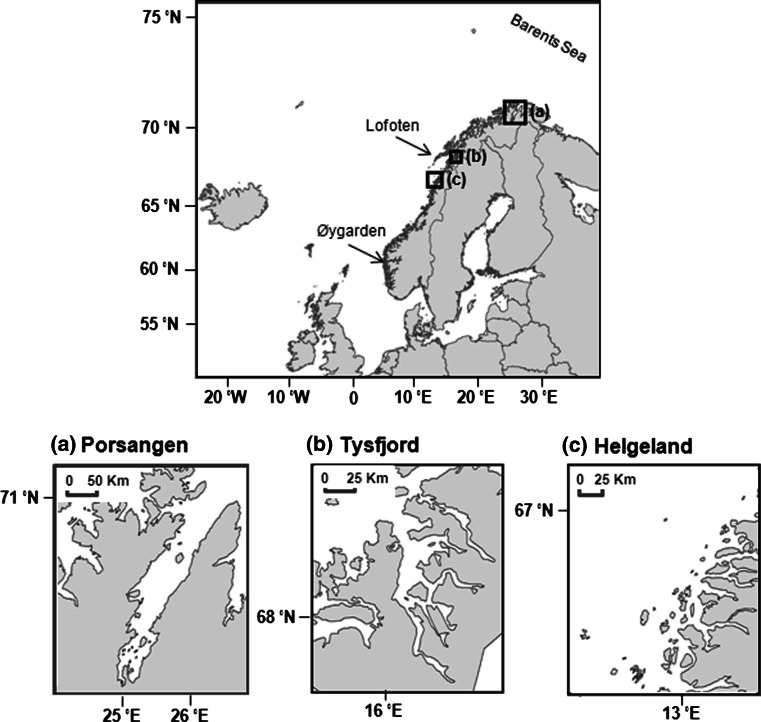
Sites of broodstock origin for two fjord-spawning populations [Porsangen (**a**) and Tysfjord (**b**)] and one coastal-spawning population [Helgeland (**c**)]. All specimens were spawned in Parisvatnet Field Station at Øygarden

Broodstocks used for the present study represent three local populations of Norwegian Coastal cod (NCC) (*Gadus morhua*) in northern Norway: two fjord-spawning populations (Porsangen fjord (in Norwegian: Porsangerfjord) and Tysfjord) and one coastal-spawning population (Helgeland) (Fig. 1). The broodstocks were born and raised in captivity from wild cod captured in the original places. The wild fish were collected in 2001–2003 as follows: fish in Porsangen fjord were collected by Danish seine in March/April 2002, and uncertain fish that might belong to the Northeast Arctic cod (NEAC) were removed based on genetic markers (Dahle et al. 2006). Tysfjord fish were taken by cod traps and hand gear in spring and autumn 2003. Fish in Helgeland region were caught by gillnets in autumn 2001. All the collected wild cod were transported to Parisvatnet Field Station of Institute of Marine Research (IMR) at Øygarden (north-west of Bergen). These wild fish were kept in captivity and allowed to naturally reproduce. The offspring that were born in 2004 were reared until adults, and in 2009 (at 5-year-old), they naturally spawned eggs used in this experiment. Thus, these eggs were the F2 generation of the wild fish. Additional information on genetics and spawning characteristics of the wild cod appears in Dahle et al. (2006) and Otterå et al. (2006), and egg buoyancy experiments done by Stenevik et al. (2008) are directly applicable to the current study for the comparison between parental and offspring generation.

To be able to categorize individual fish to family groups, all broodstocks were tagged with unique tag numbers. The fish sharing the same maternal ID (i.e. sibling relation) were regarded as a family. In this study, 5 pairs of Porsangen cod were from 3 family groups (A, B, C), 7 pairs of Tysfjord cod from 4 family groups (D, E, F, G), and 3 pairs of Helgeland cod from a single-family group (H) (Table 1). The ambient salinity for the reared cod in Parisvatnet Field Station ranged between 32 and 34. In the three original natural habitats, the salinity ranged from 34.0 to 34.8 in Porsangen, from 33.0 to 34.8 in Tysfjord and from 33.5 to 34.2 in Helgeland (Stenevik et al. 2008).

**Table 1:** Summary of genealogy, broodstock, and egg batch production for the three local Norwegian Coastal cod populations used in the point specific gravity measurements

Origin	Pair no.	Family	TL (cm)		*W* (g)		*K*		Egg batch production	
		Group	Female	Male	Female	Male	Female	Male	Total	Selected no. (PES %)
Porsangen	1*	A	71	73	7,000	5,815	1.96	1.49	10	1 (13)*
	8	B	75	82	5,800	5,850	1.37	1.06	8	4 (49), 7 (85)
	10	B	72	72	5,875	4,800	1.57	1.29	7	1 (7), 5 (66), 6 (83)
	15	B	71	70	3,400	4,940	0.95	1.44	10	4 (55), 5 (74), 10 (100)
	17	C	70	76	5,130	5,855	1.50	1.33	8	2 (39)
Mean (SD)			71.8 (1.9)	74.6 (4.7)	5,441 (1,324)	5,452 (534)	1.47 (0.36)	1.32 (0.17)	8.6 (1.3)	
Tysfjord	18	D	84	66	6,940	3,190	1.17	1.11	8	6 (74)
	22	D	81	69	7,700	4,700	1.45	1.43	10	6 (61), 7 (77)
	26*	E	88	78	8,750	5,110	1.28	1.08	10	2 (21), 3 (32), 5 (58)*
	27	F	69	73	4,300	4,650	1.31	1.20	12	8 (69)**
	28	F	67	78	4,875	5,870	1.62	1.24	12	10 (85)
	29	G	75	71	5,220	4,150	1.24	1.16	9	1 (16)
	30	G	76	73	5,710	5,260	1.30	1.35	12	3 (16), 4 (22)
Mean (SD)			77.1 (7.7)	72.6 (4.4)	6,214 (1,626)	4,704 (860)	1.34 (0.15)	1.22 (0.13)	10.4 (1.6)	
Helgeland	31	H	76	69	5,590	3,785	1.27	1.15	9	1 (12), 2 (20), 7 (78)
	32*	H	80	71	6,740	4,050	1.32	1.13	6	2 (21), 4 (69)*, 5 (88), 6 (100)
	34	H	71	76	4,750	5,300	1.33	1.21	5	5 (100)
Mean (SD)			75.7 (4.5)	72.0 (3.6)	5,693 (999)	4,378 (809)	1.31 (0.03)	1.16 (0.04)	6.7 (2.1)	

*TL* total length, *W* whole body weight, *K* Fulton’s condition factor, 100 × W/L^3^, Mean, average, *SD* one standard deviation

* Broodstock and its batch number also used for continuous specific gravity measurements

** Outlier

Total length (TL; to the nearest cm), whole body weight (W; to the nearest g), and Fulton’s condition factor (K; 100 × W/TL^3^) were measured prior to spawning. Male–female pairs were transferred to individual spawning tanks in February and monitored continuously until early May.

### Collection of cod eggs

The spawning tanks at Parisvatnet Field Station contained 1.8 m^3^ water (diameter = 1.5 m, height = 1.2 m) and showed a conical-shaped bottom. The tank colour was green, and its top was covered by a black fine-mesh net to reduce the light level by about 70 % and to prevent cod from jumping out of the tank. Water entering the tank was pumped from the neighbouring sea at a depth of 20 m and filtered through a 300 μm mesh. During spawning, water temperature was maintained at 5–6 °C, and salinity was 32–34 (flow rate: 20 L min^−1^). Egg collectors were fitted to each tank and checked daily. Eggs were naturally spawned late at night or in the early morning. Collected egg volume was measured along with mean egg diameter to calculate total number of eggs per batch (Kjesbu 1989). For a precise reference point in the spawning cycle at the time of sampling, the cumulative portion of total number of eggs spawned (PES (%); Kjesbu et al. 1990) was estimated.

### Measurement of egg specific gravity and egg diameter

Determination of egg specific gravity was performed with a density-gradient column (Martin Instruments Co. Ltd, UK) set-up in a refrigerated room (6 °C). All preparation of density gradient, calibration, accuracy, stability, and operation were as described by Coombs (1981). UV-filtered sea water, distilled water, and NaCl (ordinary salt, 99.7 %) were used to make the low and high stock solutions, which is needed to develop the density gradient in the column. Each of three columns of 80 cm height was filled by a continuously graded sea water solutions at 6 °C, and the completed density gradients ranged from 1.0160 on the surface of the column to 1.0318 g cm^−3^ on the bottom, corresponding to salinities of 20.3–40.4. At each experimental trial, five glass floats of known densities were introduced into the column from the top and ranged approximately from 1.0192 to 1.0265 g cm^−3^. The float density was originally reported at 23 °C (or 18 °C) with certified instruments in the Martin Instrument Co. (Three Crowns House, 160 Darlaston Road, Wednesbury WS10 7TA, UK). A density correction at 6 °C was obtained according to the equation based on thermal expansion: calibrated density = (float density) + (temperature difference) × (float density) × 0.000028. After 1 h, the glass floats had positioned at their levels of neutral buoyancy in the column and could be used as reference levels of specific gravity. Prepared eggs were gently inserted from the top of the column and located at their neutral buoyancies within the range of the five glass floats. The position of the glass floats was read with a precision of 1 mm and served a linear regression line between column position and density (always *R* ^2^ > 0.99). The eggs were counted every 10-mm interval 30 min after the introduction of the eggs. The egg specific gravity was derived using the regression equation (specific gravity accuracy between interval ±2 × 10^−4^).

Shortly after eggs were spawned at Parisvatnet Field Station, the collected samples of eggs was transported to the laboratory of IMR in Bergen, and kept at 6 °C in a refrigerated room until eggs developed up to the morula stage (2 days old; Fridgeirsson 1978). Specific gravity measurements progressed in two different ways: one was *continuous specific gravity measurements* over time, and the other was *point specific gravity measurements* in a given time. The former was done to track daily changes in egg specific gravity during the incubation period for the same eggs, examining one batch from each local population, as indicated in Table 1. Normally developing eggs were placed in two columns (45 ± 5 eggs for each column) at the same time for duplicate measurements. The egg specific gravity was noted on a daily basis until 50 % hatching at 24-h light conditions. Since the development at day 2 and the onset of hatching were very similar among the three studied populations under the condition of constant incubation temperature of 6 °C, we assume that eggs on the same day after fertilization had the same embryonic development in the three populations. Initially made density gradients were stable so that the glass floats positioned almost at the same level during the whole development, but for the sake of reliability, their positions were reread each time egg specific data were reported. For point measurements, only egg specific gravity at the morula stage was considered. Here, up to four batches per broodstock were used (Table 1). As always, density gradients of a column were newly made on each occasion. More than 50 eggs per batch were introduced in a single un-replicated column. Total number of egg batches used was 10 for Porsangen, 11 for Tysfjord, and 8 for Helgeland (Table 1).

After reading the position of eggs for the point measurements, all eggs were taken out of the columns with a long glass pipette to measure egg diameter. Images of the eggs were taken by a digital camera attached to a stereomicroscope at 20 × magnification. Subsequently, sharply focused images were opened in the public domain image analysis program Image J (Rasband 1997–2008), and the egg diameters (*n* = 50; to the nearest 0.001 mm) were measured and averaged for each batch.

### Data analysis

Only one statistical outlier (pair no. 27 (Tysfjord), Table 1) in egg specific gravity was identified (>3.5 standard deviations). All statistical tests were run in R version 2.10.0 (2010) and excluded the outlier due to the violation of variance homogeneity. We assessed the significance of the relationships between egg specific gravity and maternal traits and egg diameter by measuring the Pearson correlation coefficient (*r*). To meet analysis of variance (ANOVA) assumptions, that is, homogeneity of variances and normal distribution, data were log transformed, and two-way ANOVA was performed to test for significant differences in total length, whole body weight, and Fulton’s condition factor among the three local populations (1) with interaction between population and sex and (2) with interaction between population and family. Analysis of covariance (ANCOVA) was used to compare the regressions of the maternal traits across the three local cod populations on egg specific gravity. An additional ANCOVA test was performed to compare the seasonal effects (i.e. slopes) made by two to four successive egg batches on egg specific gravity. Tukey HSD test was used to examine mean differences in egg specific gravity among family groups as well as increased/decreased egg specific gravity at two egg stages (days 4 and 15) between the three local populations. To test for an effect of local population on egg specific gravity, we used a linear mixed-effect (LME) model (Pinheiro et al. 2010), with pair no. as a random effect to control for repeated measurements taken from pairs at different number of egg batches (i.e. PESs; covariate). For the effect of individual pair on egg specific gravity, in the LME model, local population was a random effect and PES was a covariate.

## Results

### Broodstock characteristics

Total length, whole body weight, and condition factor were similar among the three local populations and between females and males with no interaction (population × sex) (ANOVAs, always *p* > 0.05). However, at the family level, groups D and E had longer female total length than the other family groups (ANOVA, *F*_(5,7)_ = 8.45, *p* = 0.007), while there was no significant difference in whole body weight and condition factor among families.

### Relationships between egg specific gravity, maternal attributes, and egg diameter

**Fig. 2:**
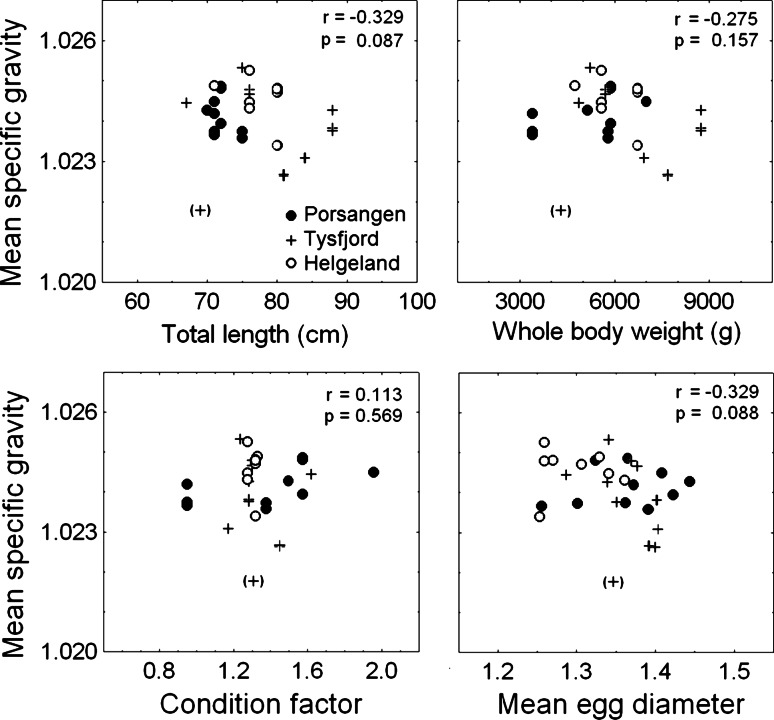
Joint* scatterplots* of mean egg specific gravity at the morula stage against maternal traits (total length, body weight, and Fulton’s condition factor) and mean egg diameter.* Points* are mean egg specific gravity of a batch corresponding to the independent variables. The* point in parentheses* is identified as an outlier. The plots display *r*, the Pearson correlation coefficient, and the corresponding *p* value when excluding the outlier

The relationships between egg specific gravity and female total length (Pearson’s correlation, *r* = −0.33, *p* = 0.09) and mean batch egg diameter (Pearson’s correlation, *r* = −0.33, *p* = 0.09) were not statistically significant, but a negative trend was noticed in both cases concurrent with a high variation (Fig. 2). Likewise, female body weight (Pearson’s correlation, *r* = −0.28, *p* = 0.16) and Fulton’s condition factor (Pearson’s correlation, *r* = 0.11, *p* = 0.57) did not correlate significantly with egg specific gravity (Fig. 2).

The slopes of the regression of egg specific gravity and female total length clearly did not differ among the three local populations (ANCOVA, *F*_(2,22)_ = 0.07, *p* = 0.93). Neither did the corresponding regression of egg specific gravity and egg diameter differ significantly (ANCOVA, *F*_(2,22)_ = 2.85, *p* = 0.08).

### Individual versus population variance in egg specific gravity

**Fig. 3:**
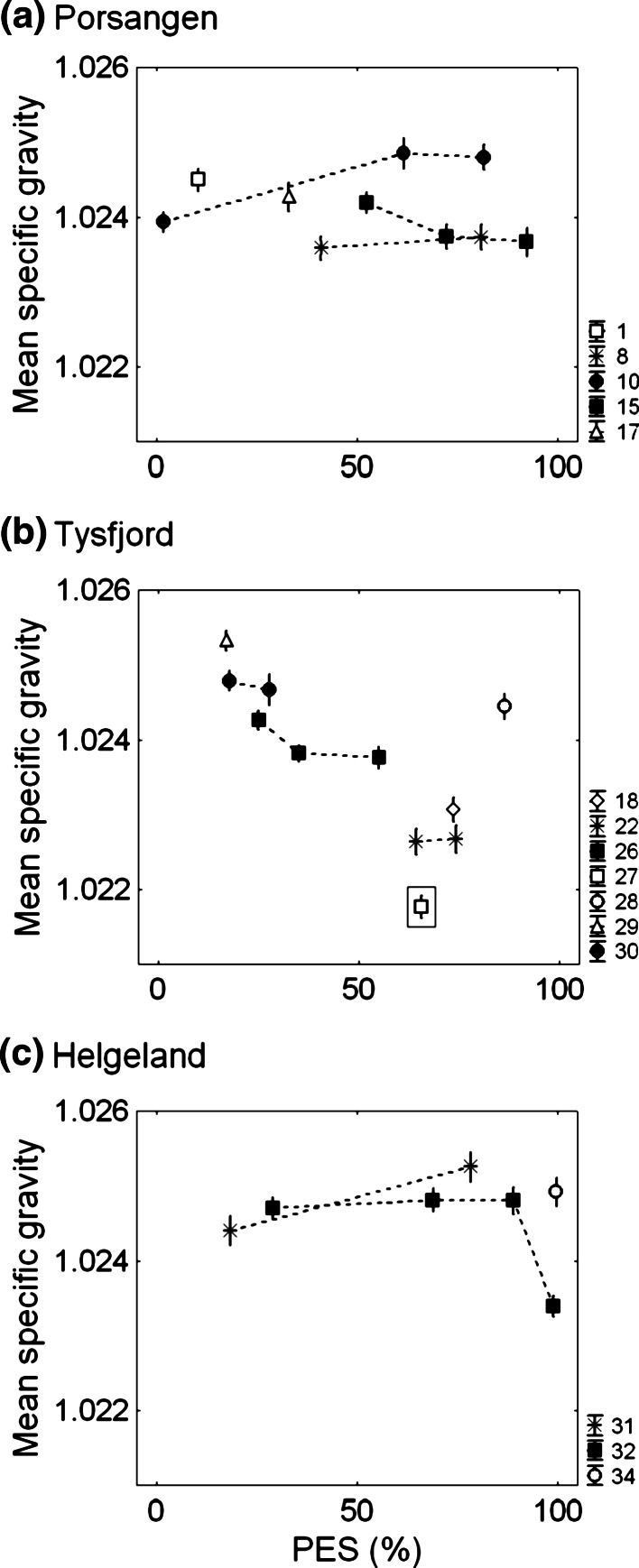
Seasonal changes in egg specific gravity at the morula stage of paired broodstocks for three local populations.* Symbol numbers* denote different pair numbers. PES is the portion of spawned eggs during the experimental period.* Vertical bars* denote one standard deviation. Data point in the* rectangular box* is an outlier

Mean specific gravity was highest for Helgeland (1.0246 g cm^−3^), intermediate for Porsangen (1.0241 g cm^−3^), and lowest for Tysfjord (1.0239 g cm^−3^) (at 6 °C) (Table 2). However, there was no evidence of any inter-population difference in phenotypes of egg specific gravity, both in the case of exclusion (LME, *n* = 1,392, *F*_(2,11)_ = 1, *p* = 0.51) and inclusion (LME, *n* = 1,442, *F*_(2,12)_ = 1, *p* = 0.36) of the mentioned outlier. In contrast, the egg specific gravity was significantly different at the individual pair level (LME, *n* = 1,392, *F*_(13,1,375)_ = 238, *p* < 0.001). Two to four consecutive egg batches did not show a general trend in egg specific gravity with portion of eggs spawned (PES) between/within populations, tested with ANCOVA slop differences (PES × Pair no., all model runs, *p* < 0.001) (Fig. 3). Variation in egg specific gravity was greatest between pairs in the Tysfjord population.

**Table 2:** Average, standard deviation (SD), minimum, and maximum of egg specific gravity for the three local Norwegian Coastal cod populations

Origin	Specific gravity				Transformation into salinity unit (ppt) at 6 °C			
	Mean	SD	Minimum	Maximum	Mean	SD	Minimum	Maximum
Porsangen (*n* = 10)	1.0241	0.00048	1.0231	1.0251	30.6	0.6	29.3	31.9
Tysfjord (*n* = 10)	1.0239	0.00089	1.0223	1.0255	30.4	1.1	28.3	32.4
Helgeland (*n* = 8)	1.0246	0.00055	1.0232	1.0258	31.2	0.7	29.5	32.7

The outlier was excluded from Tysfjord population. *N* is the total number of batches examined in each population

### Family variance in egg specific gravity

**Fig. 4:**
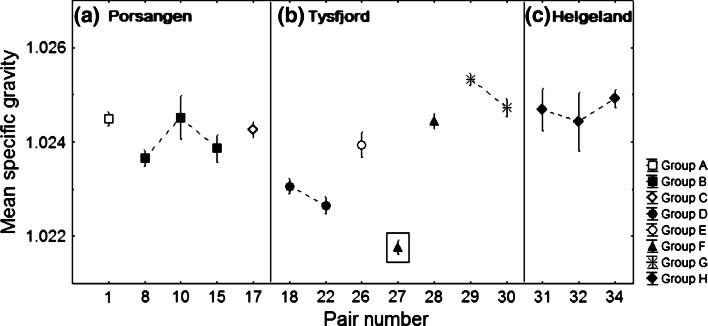
Variability of egg specific gravity at the morula stage within paired broodstocks for three local populations differentiated by family groups (**a**–**h**). Each pair has different number of egg batches according to Table 1. *Vertical bars* denote one standard deviation. Data point in the *rectangular box* is an outlier

Porsangen family groups A, B, and C expressed different phenotypes of egg specific gravity (Tukey HSD test, A ↔ B *p* < 0.001; A ↔ C *p* = 0.046; B ↔ C *p* = 0.01). Also, Tysfjord family groups D, E, F, and G showed significantly different family means of egg specific gravity (Tukey HSD test, all results *p* < 0.001). However, Helgeland analysis was limited to a single-family group (H), and the three pairs were similar in egg specific gravity (ANOVA, *F*_(1,396)_ = 0.18, *p* = 0.67) (Fig. 4).

### Incubation effect on egg specific gravity

**Fig. 5:**
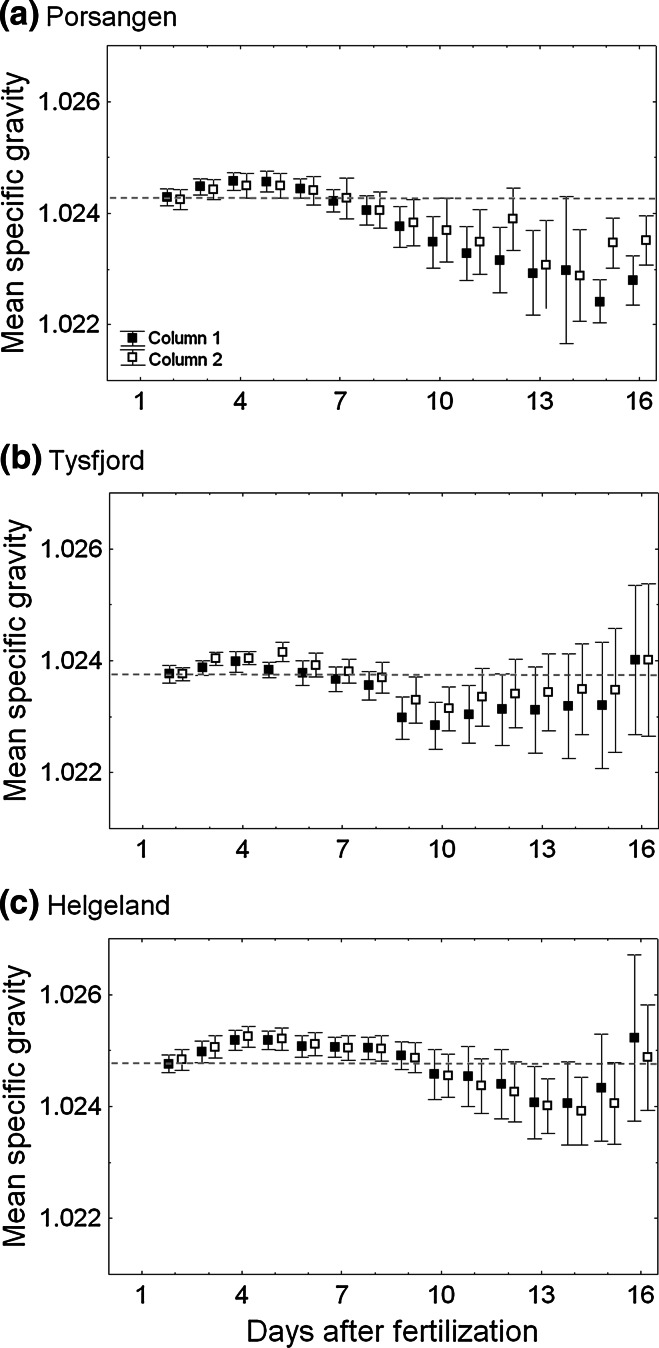
Daily changes in egg specific gravity from the morula stage (2 days old) until 50 % hatching, that is, continuous measurements, by using replicates (columns 1 and 2) for each local population. Broodstock used is pair no. 1 (1st batch), 26 (5th batch), and 32 (4th batch) for Porsangen, Tysfjord, and Helgeland, respectively. *Horizontal dotted line* denotes mean egg specific gravity of the morula stage. *Vertical bars* refer to one standard deviation

During the length of the incubation period, Tysfjord and Helgeland eggs had almost no mortality, while Porsangen eggs had 20 % cumulative mortality at 50 % hatching. Nevertheless, the pattern of temporal changes from the morula stage (2 days old) to approximately 50 % hatching (Fig. 5) was similar in the three local populations; at first, mean egg specific gravity increased slightly until gastrulation (4–5 days old) and then gradually decreased, but with an abrupt increase immediately prior to hatching. The variance of newly fertilized eggs was very narrow, but the variance increased markedly towards hatching. The slight amount of increase from day 2 to day 4 was biggest for Helgeland eggs (on average 0.00042 g cm^−3^ increase) and significantly different from Porsangen (0.00028 g cm^−3^) (Tukey HSD test, *p* = 0.008) and Tysfjord eggs (0.00022 g cm^−3^) (Tukey HSD test, *p* < 0.001). The noted increase for Porsangen and Tysfjord was similar (Tukey HSD test, *p* = 0.22). On the other hand, the gradual decrease from day 4 to day 15 was biggest for Porsangen eggs (−0.0019 g cm^−3^) and significantly different from Tysfjord (−0.0006 g cm^−3^) (Tukey HSD test, *p* < 0.001) and Helgeland eggs (−0.0004 g cm^−3^) (Tukey HSD test, *p* < 0.001). The decrease for Tysfjord and Helgeland was similar (Tukey HSD test, *p* = 0.81). Helgeland eggs were heavier than the initial specific gravity until day 9, and Porsangen and Tysfjord eggs were heavier until day 6 (Fig. 5).

## Discussion

### Factors affecting egg specific gravity

Egg specific gravity is determined by the balance between light components (e.g. water and lipid) and heavy components (e.g. salt and protein in the yolk and chorion) (Craik and Harvey 1987). For pelagic fish eggs, high water content and chorion volume are regarded as major regulators of egg specific gravity (Kjesbu et al. 1992; Thorsen et al. 1996). The water reservoir is formed during final oocyte maturation by the process of hydration (resulting in “swelling”) in which an extensive hydrolysis of ovoplasmic protein into free amino acids (FAA) occurs (Craik and Harvey 1984; Thorsen and Fyhn 1996). It is hypothesized that osmotic gradients occur between the ovarian fluid and the oocyte since the resulting osmolality is similar among female blood, ovarian fluid, and ovulated eggs (Davenport et al. 1981). Lipid is generally considered to have minor importance for egg specific gravity (Craik and Harvey 1987; Thorsen et al. 1996). Additional slight changes in osmolality occur due to the formation of perivitelline space between chorion and embryonic membrane at the time of fertilization, being filled by ambient seawater (Davenport et al. 1981).

Examples in the literature show that egg specific gravity is, in one way or the other, associated with the environment in which the fish live. For instance, in the pioneer work of Solemdal (1973), flounder (*Pleuronectes flesus*) eggs in the brackish water typically had specific gravity of 1.012, while their marine counterparts had specific gravity of 1.026. When the brackish flounder females were transferred to the marine environment, the specific gravity increased to 1.017 2 years after acclimation to water salinity of 35, that is, approached but did not reach the same egg specific gravity as the marine flounder. The difference in egg specific gravity was ascribed to the change in blood plasma osmolality. In another experimental study on spotted seatrout (*Cynoscion nebulosus*) by Kucera et al. (2002), also egg specific gravity increased with increasing spawning salinity. Thorsen et al. (1996) concluded that the ability of brackish cod eggs to increase water content during hydration may be heritable. In our experimental set-up, seawater was pumped from 20 m depth in a neighbouring coastal area to meet natural environmental changes, and temperature and salinity varied between 5 and 6 °C and between 32 and 34, respectively. A potential modification of the effect of local natural environment is expected for Porsangen fjord since cod in this area would frequently encounter water salinities somewhat higher than encountered in this study, that is, between 34 and 35 (Stenevik et al. 2008). Consequently, the average egg specific gravity of Porsangen obtained here might be underestimated, but the degree of modification is expected to be negligible.

Atlantic cod is a multiple batch-spawner, producing up to 20 egg batches within a single-spawning season, and egg size becomes progressively smaller with increasing egg batches (i.e. PES) (Kjesbu 1989). In this study, seasonal changes in egg specific gravity within individual females did not show a general pattern throughout the entire spawning season both within and between local populations. The seemingly negative trend for Tysfjord was attributable to a significant effect of individual pairs and restricted sample nos., but unlikely due to a PES effect. In addition, egg size was not significantly correlated with the egg specific gravity. However, negative trends between egg size and egg specific gravity have been found in an earlier study on Norwegian Coastal cod (NCC) (Kjesbu et al. 1992) and likewise for Baltic cod (Nissling et al. 1994; Vallin and Nissling 2000). In contrast, Marteinsdóttir and Begg (2002) found a positive correlation for Icelandic cod eggs. We could not interpret the contradictory result from Icelandic cod eggs, but there might be differences in egg composition such as water, lipid content and chorion thickness, parental condition factor, spawning experience, etc.

### Incubation effects on egg specific gravity

How egg specific gravity varies during development is still ambiguous due to the small number of adequate studies. Previous studies have created contrasting experimental results. Here, we have found a general pattern of temporal changes in egg specific gravity during embryogenesis for Atlantic cod; newly fertilized eggs showed a slight increase in specific gravity until the completion of the gastrulation (days 4–5) and then a steady decline, although with signs of an increase just before hatching. In terms of regulating mechanism, lipid (0.9 g cm^−3^) and water content are the only two upward directed forces in teleost eggs with specific gravity less than that of sea water (Craik and Harvey 1987). During development, there is apparently no significant change in water content of cod eggs, whereas egg dry mass decreases to some extent after the gastrulation (Finn et al. 1995). Consequently, changes in biochemical components will likely be the main causes that account for decreased specific gravity towards hatching. Through egg metabolism, a considerable change occurs in the content of free amino acids (FAA), which are denser than sea water and known to decrease by 50 % of initial amount right before hatching. In contrast, the other chemical components change relatively little (Fyhn and Serigstad 1987). Therefore, the contribution of lipid content to achieve positive egg specific gravity may increase during ontogeny, as FAA content decreases.

The present general pattern seen for NCC during incubation is consistent with the conspecific study of Anderson and deYoung (1994) on Northern cod where egg specific gravity decreased successively after a slight rise as found in here with a 5–9 % reduction in egg specific gravity between fertilization and hatching. In contrast, in the NCC study of Mangor-Jensen (1987), overall egg specific gravity increased gradually over time, but with a slight decrease after gastrulation. As for Baltic cod, two previous studies are not in agreement with our findings. In the study of Nissling and Westin (1991), egg specific gravity was constant during most embryonic stages, and then suddenly increased prior to hatching. On the other hand, Nissling and Vallin (1996) measured several different patterns under the same experimental set-up. Note, however, that the temporal changes in specific gravity collected from an egg batch with low mortality rates coincides exactly with our results. In addition to cod eggs, pelagic Atlantic halibut (*Hippoglossus hippoglossus*) eggs (Mangor-Jensen and Waiwood 1995) and Cape hake (*Merluccius capensis*) eggs in northern Benguela ecosystem (Sundby et al. 2001) also had a similar decreasing pattern during embryonic development. Recent finding of temporal changes in egg specific gravity of European anchovy (*Engraulis encrasicolus*) in Mediterranean (Ospina-Álvarez et al. 2012) was very similar to our result. Therefore, the pattern found in our study seems to be typical for marine pelagic eggs. One likely candidate for erroneous conclusions in various studies might be the introduction of poor egg quality (Kjørsvik et al. 1990), a topic which is not pursued further here.

### Are there local population differences in egg specific gravity?

The previous study of Stenevik et al. (2008) on wild-caught NCC populations from the same origins found particularly light eggs from Porsangen, heavy eggs from Tysfjord, and intermediate Helgeland eggs, indicating statistically significant differences in egg specific gravity. However, the present more comprehensive current study did not find any such differences. The reason for this can be fewer egg batches and broodstocks in the study of Stenevik et al. (2008). Therefore, considering the larger variability of egg specific gravity among individual pairs and family groups rather than populations, it is assumed that local populations can have more diverse phenotypes of egg specific gravity in nature when originating from more families and individual pairs.

**Fig. 6:**
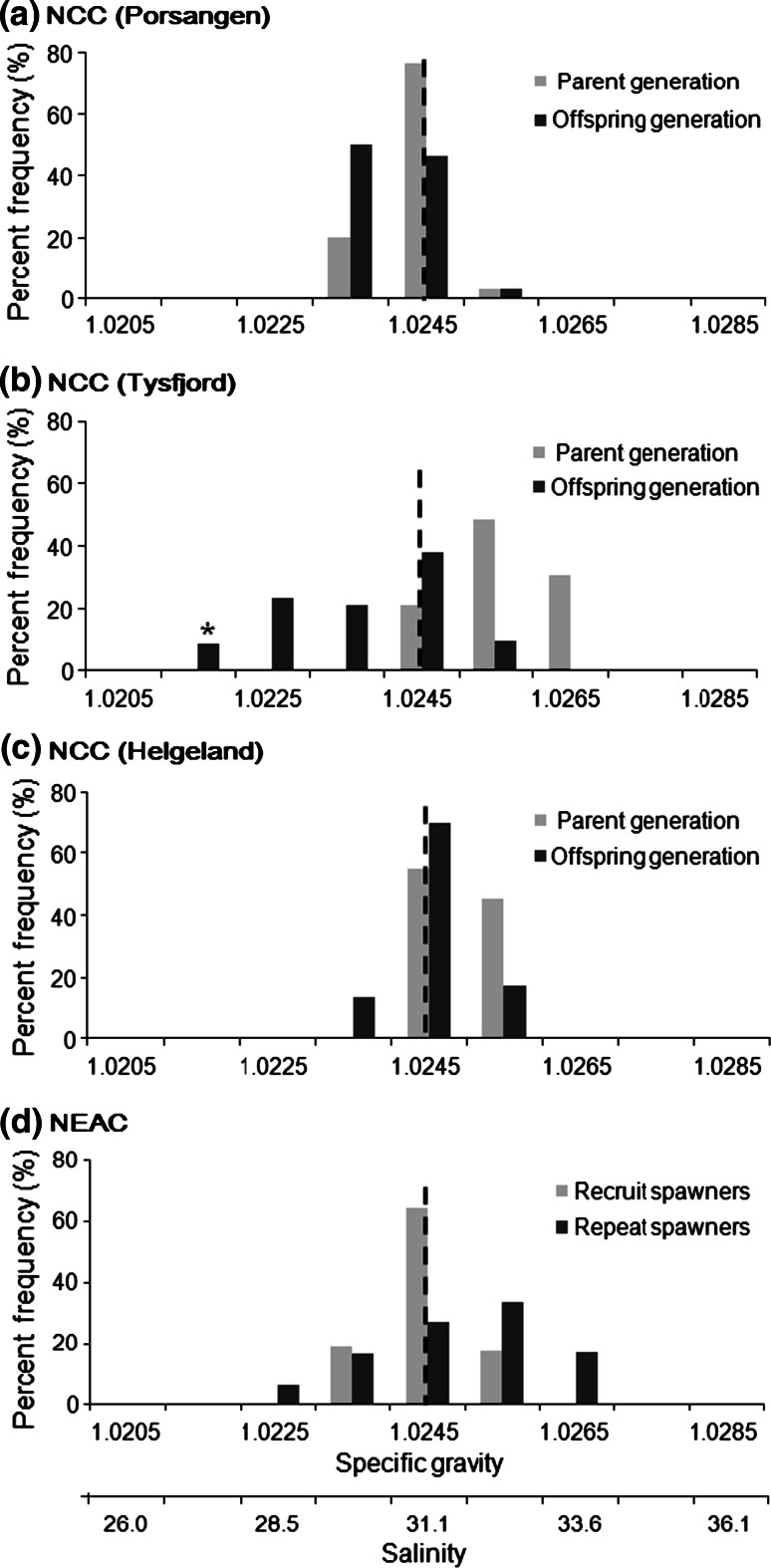
Egg specific gravity comparison between Norwegian Coastal cod (NCC; Porsangen, Tysfjord, Helgeland) and Northeast Arctic cod (NEAC). NEAC egg specific gravity was adjusted to that of the morula stage by using the per cent difference (cf. Fig. 5) from coastal-spawning cod (Helgeland). Data of NEAC and parent NCC generation were provided by Kjesbu OS (unpubl data) and Stenevik et al. (2008), respectively. *Vertical dotted lines* refer to the combined mean specific gravity in each for all combined geographical population.* Asterisk* is an outlier

In the following comparison between parental and offspring generation (Fig. 6), Tysfjord showed a significantly different egg specific gravity between parental and offspring generations (ANOVA, *F*_(1,40)_ = 12.85, *p* = 0.001), whereas Porsangen and Helgeland had similar egg specific gravities between the two generations (ANOVA, *F*_(1,22)_ = 2.13, *p* = 0.16). However, it must be noted that Tysfjord eggs of the parental generation averaged 1.31 mm in diameter (median 1.29 mm), which is not only the smallest size measured between local populations of the parental generation but also a lower value than seen for the offspring (mean, 1.36 mm; median, 1.37 mm) (data not shown). Based on the negative egg diameter–egg specific gravity relationship observed and modelled by Kjesbu et al. (1992), heavier eggs thus can be expected for the smaller egg size of the Tysfjord offspring group.

Recent genetic studies suggest not only a small-scaled geographical differentiation of local cod populations across continuous Norwegian coastal regions (Knutsen et al. 2003; Jorde et al. 2007) but also population differences between inshore and offshore waters (Westgaard and Fevolden 2007). Dahle et al. (2006) found a minor but significant genetic difference between the Tysfjord and Helgeland populations. The findings of a high degree of site fidelity of adult cod (Jakobsen 1987; Espeland et al. 2007) and a high retention mechanism of cod eggs inside fjords (Ciannelli et al. 2010; Myksvoll et al. 2011) make such a small-scale population structure more reasonable. In this sense, we tested whether or not local cod populations evolve inherent egg specific gravity adapted to the local environments in regard to population connectivity during egg stages. However, in the present analysis, the phenotype of egg specific gravity seems to be generally similar among local populations, suggesting that local environmental conditions play a key role in the maintenance of local population structures by affecting egg distributional patterns. Previous model simulations of vertical distribution of cod indicated that the local ambient salinity profile, and, consequently, the specific gravity of the ambient sea water is more important for egg vertical distribution than potential differences in the specific gravity of the eggs (Stenevik et al. 2008). This fact became even clearer and more specific in the modelling of NCC in Folla by Myksvoll et al. (2011), a neighbour fjord of Tysfjord. Accordingly, a likely adaptation mechanism for establishing the local cod populations is that spawners by selecting specific spawning sites in the fjords with brackish water in the surface layer strongly impact the retention of the eggs as they become influenced by the concentration mechanism of the estuarine circulation (Myksvoll et al. 2011). The conclusion in the present paper that egg specific gravity among the coastal cod stocks is not significantly different implies that the various populations have not to a varying extent developed egg specific gravities particularly adapted to the local fjord habitat. The local salinity structure will determine whether the eggs be retained or dispersed out of the fjord. This further points to that vagrant cod that might happen to spawn in a neighbour fjord will have their eggs and larvae retained in the fjord together with the eggs from the local population. Consequently, the only way to maintain separate populations between the fjords is that the mature fish actively seeks back to the sites they were born, that is, natal homing.

### NCC and NEAC separation mechanisms

In mid and northern Norway, NCC spawn partly at the same spawning grounds as the NEAC (Ottersen and Sundby 2005). Processes differentiating the two stocks during early life history have not yet been fully understood. One study of Løken et al. (1994) clarified that NCC fry settles at shallower depths and earlier in the season than that of NEAC, which suggests that the different settling strategy for young cod might prevent NCC from mixing with NEAC. Modelling studies also indicate markedly different drift trajectories depending on offspring vertical distribution (Vikebø et al. 2007). Thus, it is of great interest to examine whether NCC and NEAC have a different egg buoyancy strategy in the same spawning area or not. Fortunately, we do have access to comparable egg specific gravity data from wild broodstocks captured in the south-western part of the Barents Sea (Fig. 6; Kjesbu OS, unpubl. data). The specific gravity of these NEAC eggs were similar to those reported by Solemdal and Sundby (1981) where the range of egg specific gravity of stripped and artificially fertilized eggs in Lofoten was 1.023–1.026 g cm^−3^ (at 5 °C). From the comparison of egg specific gravity between NCC and NEAC, an extensive overlap appears between coastal-spawning cod (i.e. Helgeland) and NEAC. However, one should not overlook the incubation effect on egg specific gravity, since the reduction in egg specific gravity over the time of development was different among the three local populations (Fig. 5). Therefore, if NEAC eggs have a high reduction in initial specific gravity at later stages, the NEAC eggs would be more advected northwards by offshore current transport than NCC eggs.

## Conclusion

We have found that the phenotype of egg specific gravity was similar among local Norwegian Coastal cod populations and that the specific gravity variability was greater at the individual level than at the population level. As a result of our study, at the same spawning areas, the eggs from the two stocks, NCC and NEAC, should coexist locally with the similar vertical distributions, but with some caution for temporal differences in egg specific gravity during further development. The three local populations exhibited a similar incubation effect on egg specific gravity from fertilization until hatching, even though the reduction in specific gravity was different. This indicates that a gradual decline in egg specific gravity after the gastrulation stage is a typical pattern for Atlantic cod populations.
